# Perception of Water-Based Masking Sounds—Long-Term Experiment in an Open-Plan Office

**DOI:** 10.3389/fpsyg.2017.01177

**Published:** 2017-07-18

**Authors:** Valtteri Hongisto, Johanna Varjo, David Oliva, Annu Haapakangas, Evan Benway

**Affiliations:** ^1^Finnish Institute of Occupational Health Turku, Finland; ^2^Turku University of Applied Sciences Turku, Finland; ^3^Independent Researcher Turku, Finland; ^4^Plantronics, Inc. Santa Cruz, CA, United States

**Keywords:** open-plan offices, acoustics, masking, noise, noise annoyance, environmental psychology, environmental satisfaction

## Abstract

A certain level of masking sound is necessary to control the disturbance caused by speech sounds in open-plan offices. The sound is usually provided with evenly distributed loudspeakers. Pseudo-random noise is often used as a source of artificial sound masking (PRMS). A recent laboratory experiment suggested that water-based masking sound (WBMS) could be more favorable than PRMS. The purpose of our study was to determine how the employees perceived different WBMSs compared to PRMS. The experiment was conducted in an open-plan office of 77 employees who had been accustomed to work under PRMS (44 dB *L*_Aeq_). The experiment consisted of five masking conditions: the original PRMS, four different WBMSs and return to the original PRMS. The exposure time of each condition was 3 weeks. The noise level was nearly equal between the conditions (43–45 dB *L*_Aeq_) but the spectra and the nature of the sounds were very different. A questionnaire was completed at the end of each condition. Acoustic satisfaction was worse during the WBMSs than during the PRMS. The disturbance caused by three out of four WBMSs was larger than that of PRMS. Several attributes describing the sound quality itself were in favor of PRMS. Colleagues' speech sounds disturbed more during WBMSs. None of the WBMSs produced better subjective ratings than PRMS. Although the first WBMS was equal with the PRMS for several variables, the overall results cannot be seen to support the use of WBMSs in office workplaces. Because the experiment suffered from some methodological weaknesses, conclusions about the adequacy of WBMSs cannot yet be drawn.

## Introduction

Noise and lack of acoustic privacy are typically the most adverse factors of work environment in open-plan offices (Pejtersen et al., 2006; Haapakangas et al., 2008; Bodin Danielsson and Bodin, 2009; Frontczak et al., 2012). Coworkers' speech is usually the most annoying noise source (e.g., Banbury and Berry, 2005; Kaarlela-Tuomaala et al., 2009; Pierrette et al., 2015; Hongisto et al., 2016a).

Evidence from experimental psychology suggests that irrelevant speech, i.e., background speech that is not useful to the performed task, has detrimental effects on cognitive performance (e.g., Martin et al., 1988; Salamé and Baddeley, 1989; Hongisto, 2005; Schlittmeier et al., 2008; Haka et al., 2009). The effects are not related to the sound pressure level (*SPL*) of speech but to the speech intelligibility (Colle, 1980; Ellermeier and Hellbrück, 1998; Hongisto, 2005; Schlittmeier et al., 2008; Hongisto et al., 2016b). Therefore, the room acoustic design of open-plan offices should aim at reducing intelligibility of speech beyond the distance where normal conversations are carried out (Hongisto et al., 2004; Virjonen et al., 2009; Keränen and Hongisto, 2013).

Speech intelligibility can be objectively estimated in offices by the Speech Transmission Index, *STI*, which can have values from 0.00 (no intelligibility) to 1.00 (perfect intelligibility). The measurement of *STI* has become an international practice instead of reverberation time in open-plan offices after 2012 (ISO 3382-3, 2012). Hongisto (2005) has suggested that *STI* values below 0.20 might be low enough to avoid the negative effects of background speech on cognitive performance. Various experiments have supported or partially supported this view (Haka et al., 2009; Jahncke et al., 2013; Haapakangas et al., 2014; Keus van de Poll et al., 2014; Ebissou et al., 2015; Schlittmeier and Liebl, 2015; Hongisto et al., 2016b).

A recent study including 21 offices provides evidence that noise disturbance is lower in offices where the distraction distance is smaller (Haapakangas et al., 2017). Distraction distance is an objective single-number quantity which describes the room acoustic quality of an open-plan office. It is measured according to ISO 3382-3 (2012). It expresses the distance, in meters, from a speaker where the *STI* falls below 0.50. The smaller the distraction distance is, the smaller is the floor area that a single speaker disturbs in the office space. The finding is very important because it provides strong evidence that investing in room acoustic quality can be profitable in a wide perspective. Because individual workplaces differ strongly in activity noise levels and work tasks, a causal link between distraction distance and noise disturbance may be difficult to find.

The reduction of *STI* and distraction distance can be achieved in open-plan offices by the simultaneous application of strong room absorption, adequate level and spectrum of sound masking (Virjonen et al., 2009; Keränen and Hongisto, 2013) and high screens surrounding the workstations. Based on abovementioned references, it is relatively easy to conclude and demonstrate that the increment of sound masking level is the easiest way of reducing the *STI*. Furthermore, Haapakangas et al. (2017) found that noise disturbance was lower in offices where the background noise level was higher. Their study involved background noise levels between 29 and 45 dB *L*_Aeq_, where *L*_Aeq_ is the A-weighted equivalent sound pressure level. A-weighting is a standard method of expressing the sound level of sound using a single number. It takes the human hearing sensitivity for frequencies 20–20,000 Hz into account. It should be noted that masking levels above 45 dB are not recommended because they can lead to raised speech effort and do not increase the desired benefit of masking sound (Veitch et al., 2002; Bradley, 2003).

**Figure 1:**
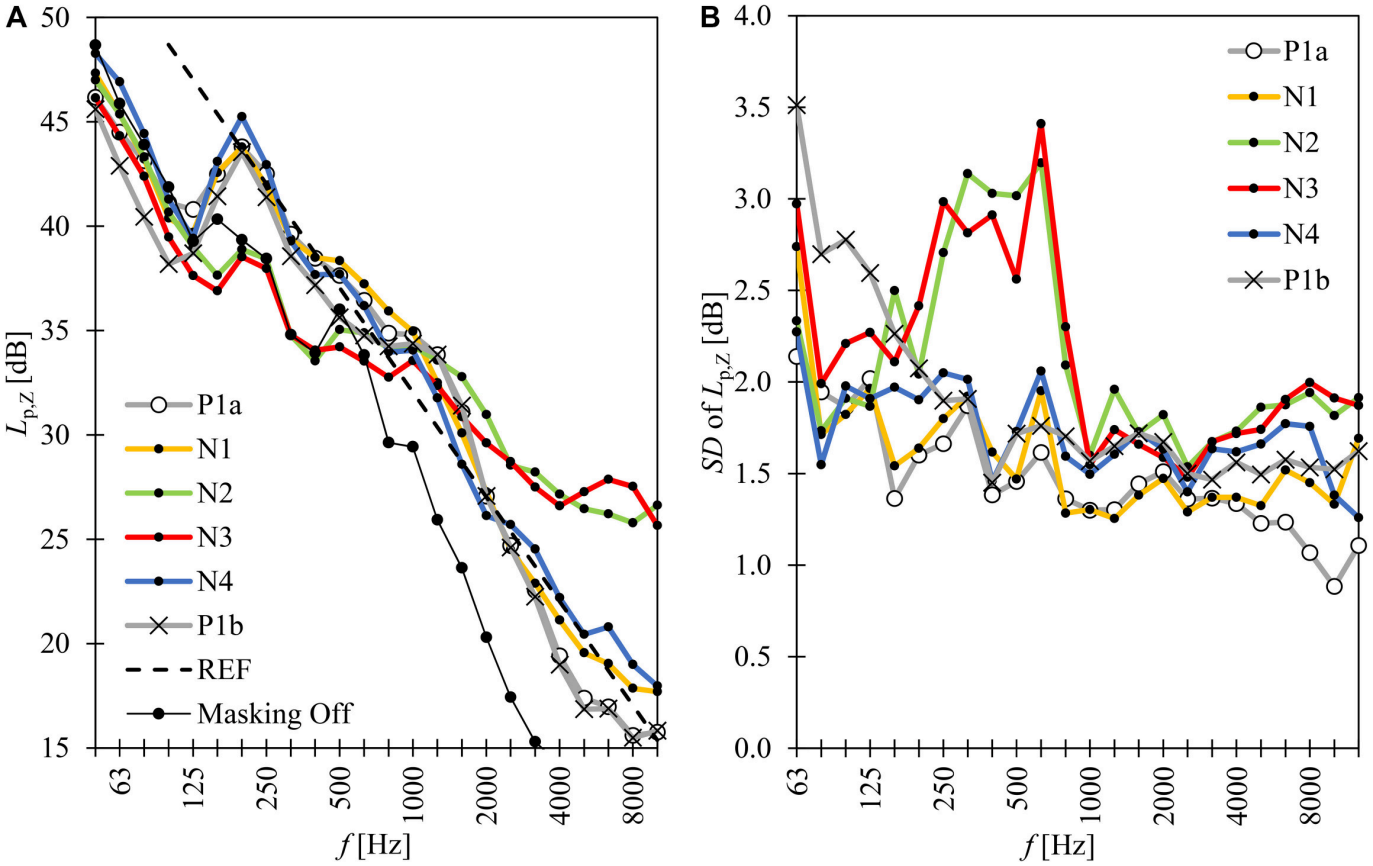
The measured spectra of the masking sounds over the 54 workstations. **(A)** Mean value and **(B)** standard deviation (*SD*). REF represents the slope −5 dB per octave doubling at a level of 44 dB *L*_Aeq_. Masking Off corresponds to ventilation noise.

The positive effects of steady-state sound masking on cognitive performance and the disturbance of background speech have been observed in several laboratory experiments (see a short review of Hongisto et al., 2016b). These experiments involved nearly similar sound masking spectra, which had a slope of −5 dB per octave doubling within 125 and 8,000 Hz (curve REF in Figure 1A). There are also some field studies where the effects of room acoustic refurbishments, such as sound masking, on employees' perceptions were investigated before and after the refurbishment (Warnock, 1973; Keighley and Parkin, 1979; Lewis et al., 2003; Helenius and Hongisto, 2004; Hongisto, 2008; Hongisto et al., 2012; Vassie and Richardson, 2017). These studies give quite contradicting impression on the perception of sound masking. The main reasons are the differences in research methodology, type of masking sounds (spectra, levels), playback (headphones or loudspeakers) and control over the level. The refurbishments described by Helenius and Hongisto (2004), Hongisto (2008) and Hongisto et al. (2012) were followed by some positive changes in subjective outcomes, such as the disturbance caused by colleagues' speech. However, these studies lacked a control group so the causal effect of sound masking on subjective outcomes could not be suggested.

Sound masking is increasingly used in open-plan offices, and also in meeting and smaller office rooms, to reduce the distractions caused by irrelevant speech and to improve the speech privacy of confidential communication. Filtered pseudo-random noise, such as brown noise, is commonly applied as the sound masking signal because it is cheap to produce (royalty free) and it resembles ventilation noise. The steady-state nature of brown noise facilitates the habituation to the sound. Such a sound masking spectrum has been investigated in field conditions by, e.g., Helenius and Hongisto (2004), Hongisto (2008) and Hongisto et al. (2012). A laboratory study of Hongisto et al. (2015) showed that the subjects preferred a pseudo-random noise with a slope of −7 dB per octave doubling to a slope of −5 dB per octave doubling. Their study showed that the spectrum is an important basic feature of sound masking that should be carefully reported when the perception of sound masking is investigated.

The masking sound does not need to originate from a pseudo-random noise generator. Desirable masking spectra can be produced by various sound sources, such as music or natural sources. Employees' preferences for these alternative masking sound sources, which are not based on pseudo-random noise, have been very little investigated because few employers accept experimental studies in their workplaces. Haapakangas et al. (2011) found that a pouring water sound was a better speech masker with respect to acoustic satisfaction and cognitive performance than vocal music, instrumental music, pseudo-random noise or ventilation, although all of these sounds had exactly the same equivalent A-weighted *SPL* (45 dB) and spectrum slope (−5 dB per octave doubling). The cognitive and subjective benefits of a water sound have since been supported by Keus van de Poll et al. (2015).

Commercial sound masking systems use mainly pseudo-random noise, such as brown noise. Pseudo-random noise is very monotonous and free from noticeable temporal fluctuations. The spectrum can be shaped to resemble a typical ventilation noise and, thus, the sound may be easily accepted in buildings already involving a mechanical ventilation system. Water sounds of various kinds have been found to be favorable maskers of, e.g., road traffic noise in urban environments (Watts et al., 2009; Jeon et al., 2010, 2012; You et al., 2010). Water-based sound masking might be more preferable than pseudo-random noise in office workplaces where natural elements, such as photos of nature, plants, views to the nature and natural colors, are often used to improve environmental satisfaction. Natural elements have also been found to improve restoration after office work (Jahncke et al., 2011). Haapakangas et al. (2011) suggested that field research should be conducted to confirm their finding regarding the preference of water-based sound masking. To our knowledge, such field studies have not been published.

The purpose of our study was to determine how employees perceive different water-based masking sounds (WBMSs) compared to pseudo-random masking sound.

## Materials and methods

### Study design

**Figure 2:**
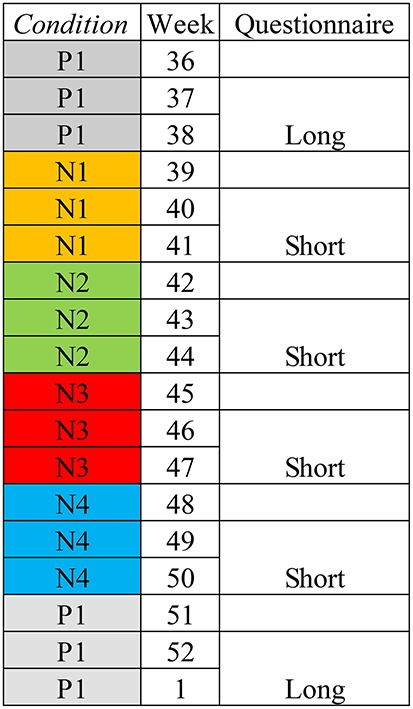
The duration of the experiment was 18 weeks. The length of the questionnaire for each *condition* is indicated.

An experiment involving a predefined order of experimental conditions was conducted at a single workplace. The independent variable is the *condition*. Five *conditions* (Table 1) involved the manipulation of the masking sound in the whole office. The duration of each *condition* was 3 weeks (Figure 2). Dependent variables are the subjective responses obtained by questionnaires during each *condition*. All employees of the workplace were invited to participate in the study. The first was repeated in the end of the experiment so that the last *condition* could be regarded as a control. The experiment was executed from 2 September 2013 to 24 January 2014.

**Table 1:** The descriptions of the sound conditions and the mean and the standard deviation (SD) of equivalent A-weighted sound pressure level, *L*_Aeq, 15s_, based on measurements in 54 workstations.

***Condition***	**Description of masking sound**	**Mean of *L*_Aeq_ [dB]**	**SD of *L*_Aeq_ [dB]**
P1a	Pseudorandom noise, resembles a table fan	44.5	1.1
N1	Waterfall, spectrum close to P1a	44.6	1.1
N2	River	43.8	1.6
N3	Babbling river	43.0	1.6
N4	River and occasional weak bird sounds	44.2	1.3
P1b	As P1a	43.7	1.4

### Information given to the employees

The employees were not involved with the experimental design. The employees were orally informed 2 weeks before the first questionnaire about the forthcoming changes in the sound masking and about the related questionnaire surveys. The purpose of the study was explained as follows: “*The purpose of the study is to understand how comfortable you are with the level of noise in the office and to understand how office noise affects your performance and well-being at work. The results will be used in Plantronics to understand how office noise considerations can be incorporated into product offerings*.”

### Ethical considerations

The study was carried out in accordance with the guidelines of the national ethical principles (National Advisory Board on Research Ethics, 2009).

### The office

**Figure 3:**
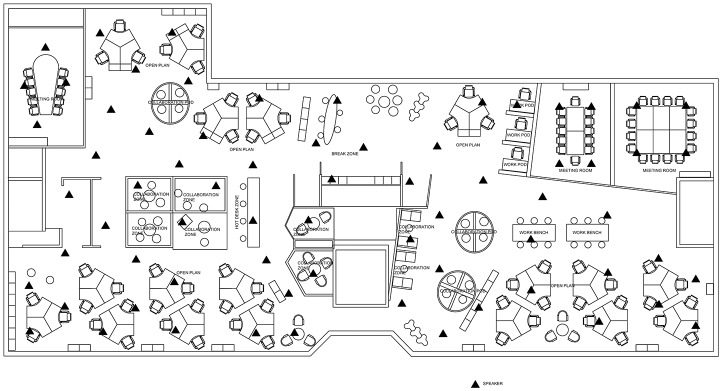
The layout of the studied open-plan office. The office involves 54 fixed workstations (18 trefoils) and more than 30 seats in collaboration zones and pods. Triangles indicate the position of masking loudspeakers.

**Figure 4:**
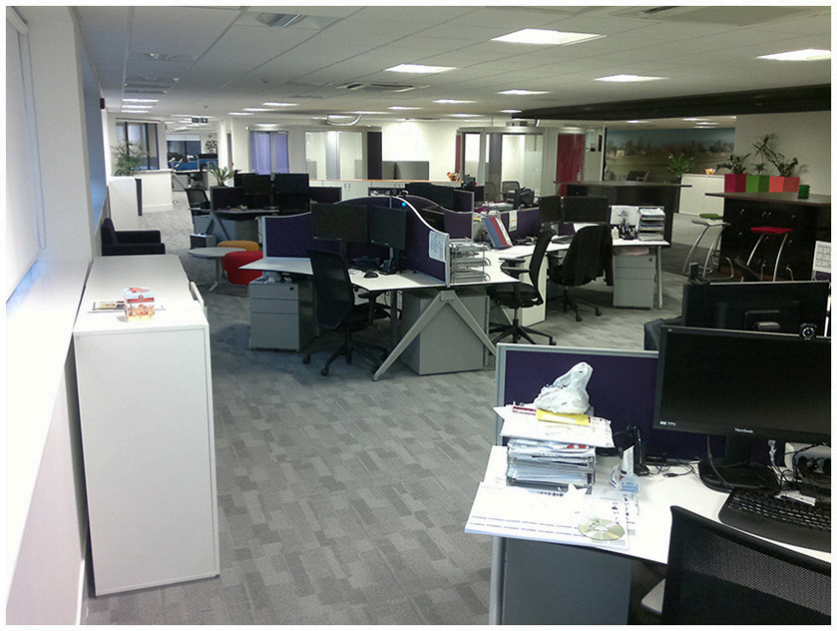
A photograph of the open-plan office. The photo was taken from the right-hand bottom corner of Figure 3.

The study was conducted at Plantronics Ltd., Royal Wootton Bassett, Swindon, Great Britain. The dimensions of the open-plan office were 19 × 50 m, altogether 930 m^2^. The office involved 54 fixed workstations. The office was occupied by 77 workers. Twenty-five of them were either traveling most of the time or worked part-time and did not have fixed workstations. The departments were: administration, IT, Quality, Sales and Marketing. The workstation areas were supported by several pods for private and communicative tasks, open meeting lounges and three large meeting rooms (Figures 3, 4).

The office had been occupied for 2 years during which PRMS (*condition* P1a) had been applied. We were told that the employees were satisfied with PRMS so a condition without any artificial masking sound was not considered realistic to test. Lack of masking was expected to increase the disturbance caused by colleagues' speech, which was not the purpose of the study.

The office was not randomly assigned but it was suggested by the funder to be a place of the experiment. The researchers found the office adequate for the study because of sufficient number of employees, existence of a sound masking system and employees' prior experience on sound masking.

### Room acoustic characterization

The room height was 3.4 m. A suspended ceiling at 2.4 m height involved sound absorbing boards (600 × 600 mm, class A, ISO 11654, 1997) in an area of 80% of the ceiling. The rest of the ceiling area involved luminaires and ventilation supplies sizing 600 × 600 mm. The floor was covered with textile carpet so walking noises were minimized and acoustic absorption area was also increased. Large wall absorber fields were installed close to the walls of break room areas. The screens between fixed workstations were standing on the tables being 1.25 m high from the floor. There was good visibility over the whole office area (Figure 4).

The room acoustic measurements were conducted according to ISO 3382-3 (2012) in the baseline *condition* (P1a). The standard describes three main quantities to be reported from the measurements: distraction distance, *r*_D_ [m], the spatial decay rate of A-weighted speech, *D*_2,S_ [dB], and the A-weighted speech level at a distance of 4 m, *L*_A, S, 4m_ [dB]. The quantity *r*_D_ expresses the distance within which a normal effort speech of a single speaker in the office remains fully intelligible and is expected to elicit full disturbance. The smaller the value is, the better is the speech privacy. Typical values lie within 3 and 18 m (Hongisto et al., 2012; Keränen and Hongisto, 2013). The distraction distance, *r*_D_, was defined in the Introduction. The quantity *D*_2,S_ expresses how many decibels the A-weighted level of speech reduces when the distance to the speaker is doubled. The larger the value is, the stronger is the attenuation of speech. Typical values are within 4 and 12 dB (Keränen and Hongisto, 2013). The quantity *L*_A, S, 4m_ describes the interpolated A-weighted level of normal effort speech at a nominal distance of 4 m from the speaker. The smaller the value is, the stronger is the attenuation of sound close to the speaker. Typical values lie within 44 and 56 dB (Keränen and Hongisto, 2013).

Sound masking (P1a) was on and the space was unoccupied during the room acoustic measurements. In the baseline *condition* (P1a), the values of the three quantities were *r*_D_ = 4 m, *D*_2,S_ = 7.0 dB and *L*_A, S, 4m_ = 45.3 dB. The speech privacy was very good from the objective point of view, because the value of *r*_*D*_ was under 5 m: the result fulfills the best class A of a Finnish guideline (RIL 243-3, 2008). The reason was adequate sound masking level, large amount of room absorbers and large room width which reduces proportion of horizontal reflections.

### Masking sounds and sound measurements

The experiment consisted of five *conditions* described in Table 1 and Figure 1. The baseline condition was repeated in the end.

The employees were accustomed to work with sound masking for the 2 years preceding the experiment. The baseline *conditions* P1a and P1b represent this original sound masking. It was produced by a pseudo-random noise generator being steady-state and broad-band. The WBMSs N1–N4 used in the actual experiment in the open-plan office were chosen after a headphone-based listening test involving altogether 16 water-based sounds. In each of them, the water flow had a very constant nature so that temporal variations were negligible. The sounds were obtained from the internet. Ten experts from the authors' organizations participated in the test via the internet. Each sound was followed by nine questions assessing the nine attributes of sound masking which were also used in the actual field experiment (Table 2). The criteria for the selection of N1–N4 were that they were sufficiently different from each other (type of water flow) and that the sounds received high satisfaction ratings in the listening test. In addition, one of the WBMSs, N4, was the same sound as in the study of Haapakangas et al. (2011). N4 involved also occasional silent bird sounds but the water sound was dominating being responsible for speech masking.

**Table 2:** Subjective measures.

**Variable name (abbreviation)**	**Item in exact form**	**Response scale**
*Acoustic satisfaction*	How satisfied are you with the office noise and acoustic environment of the office as a whole?	A
*Adequacy of masking level*	Would you like the sound level of the current sound masking to be…	B
**Perceived disturbance by environmental factors**	In the time you have spent in the office during the past month, how much have you been negatively affected by the following environmental **factors** at the office?	
*Draught*	Draught	C
*Cold*	Cold temperature	C
*Hot*	Hot temperature	C
*Stuffy air*	Stuffy air	C
*Noise*	Noise	C
*Lack of speech privacy*	Lack of speech privacy (the feeling that others can overhear what you are saying)	C
*Lighting*	Too strong or inadequate lighting	C
*Glare or reflections*	Glare of the sun or reflections on the computer display	C
*Dust or dirt*	Dust or dirt	C
*Smells*	Unpleasant smells	C
*Disorder*	Disorder at the work site (personal belongings, dishes, or papers left behind, etc.)	C
*Crowdedness*	Crowdedness at the office space	C
*Movements in vision*	Movements in the field of vision (such as other people)	C
*Openness*	Openness of the desk area, lack of screens	C
**Perceived disturbance of work by sound sources**	In the time you have spent in the office during the past week, how much have the following **sounds** disturbed your work in the office?	
*Nearby speech*	Talking and laughing in other desks	C
*Remote speech*	Talking in jointly used spaces such as collaboration and coffee areas, passage areas	C
*Ventilation*	Ventilation and air-conditioning	C
*Masking*	Sound masking (background sound coming from the office ceiling)	C
*Work sounds*	Work-related sounds generated by others such as use of keyboards	C
*Equipment*	Jointly used office equipment (such as copying machine)	C
*Phones*	Phones ringing	C
**Perceived effect of sounds on performance**	In the time you have spent in the office during the past week, how much have workplace sounds negatively affected your performance in the following **tasks**?	
*Reading*	Reading and writing	C
*Planning*	Planning and problem-solving	C
*Phone conversations*	Telephone conversations	C
*Telemeetings*	Telemeetings or phone meetings at the desk	C
*Cooperation*	Cooperation and discussions with other employees	C
*Confidential discussions*	Confidential discussions	C
**Attributes of sound masking**	There is a sound masking system operating right now in your office ceiling to provide constant background masking. Please rate, how well the following **expressions** describe your experience of the current sound masking. The sound masking is…	
*Easy to habituate to*	Easy to habituate to	D
Distracting^*^	Distracting^*^	D
*Pleasant*	Pleasant	D
Stressful^*^	Stressful^*^	D
*Natural*	Natural	D
Annoying^*^	Annoying^*^	D
Tiring^*^	Tiring^*^	D
*Acceptable*	Acceptable	D
*Helpful for my work*	Helpful for my work	D
**Open question**	Do you have other comments about the current sound masking?	
*Stress*	Have you felt stressed by your work during the past week?	E
Possibilities to influence^*^	Can you influence matters at your workplace that concern you?	E
*Support*	Do you get help and support from your colleagues when you need it?	E
*Job satisfaction*	How satisfied are you with your work as a whole?	A

Asterisk ^*^ notifies a negative attribute.

All masking sounds were produced by the masking system installed at the office (Figure 1). Seventy loudspeakers were placed above the suspended sound-absorbing ceiling, thus being hidden from the employees. Each masking loudspeaker served a floor area of approximately 14 m^2^. The effect of the transmission chain from the input signal, fed to the pre-amplifier of sound masking system, to the *SPL* measured in workstation area was determined by feeding pink noise to the masking system and by measuring the *SPL* in the workstation area. It was expected that the transmission chain was not linear because of loudspeakers' frequency response, transmission loss through the ceiling and office reverberation had complicated frequency behaviors. The measurement revealed that the speakers were unable to reproduce sounds under 125 Hz and that the transmission chain involved strong reduction of sound level at high frequencies. Therefore, the sound files N1–N4 were pre-filtered to counter-eliminate the above-mentioned transmission function.

Every time a new *condition* was launched, the adequacy of the pre-filtering (correct level and spectrum) was checked by precision measurement of *SPL* (BandK 2144 analyzer, BandK 4165 microphone) in a small office desk area (1/3-octaves from 50 to 10,000 Hz). After the final level adjustment, the spectra were measured with the same apparatus in all 54 fixed workstations and 22 other positions after which the *condition* was launched. The measurement time was 15 s per position. The launching was made during weekends when the employees were absent.

The background noise of the ventilation system (masking was off) produced a constant background noise of 40 dB *L*_Aeq_ (Figure 1). The third-octave band levels were at least 3 dB under the masking sound within 160–10,000 Hz. The ventilation noise was the dominating sound below 160 Hz. However, the ventilation noise levels were under 50 dB below 160 Hz. They were nearly inaudible, when the masking sound was turned on.

### Questionnaire

The employees were invited to respond to six questionnaires. The first and the last questionnaires were slightly longer than the questionnaires used in *conditions* N1–N4. The variables and response scales of the short questionnaire are reported in Tables 2, 3, respectively. The longer questionnaire included background information which needed not to be repeated after every condition, such as the use of the office space, nature of work, job satisfaction, stress, and education. The questionnaire was filled between the 7th and 15th day of exposure so that most employees had at least some experience on working under the current masking sound.

**Table 3:** Response scales of Table 2.

**Response scale**	**Description**
A	−2 Very dissatisfied, −1 Fairly dissatisfied, 0 Neither satisfied nor dissatisfied, +1 Fairly satisfied, +2 Very satisfied
B	−2 Much more quiet, −1 A little more quiet, 0 The same as now, +1 A little louder, +2 Much louder
C	1 Not at all, 2 Slightly, 3 To some extent, 4 Quite a lot 5 Very much
D	−3 Strongly disagree …+3 Strongly agree (7 point 2-pole scale, only extreme values were verbally labeled)
E	1 Not at all, 2 Slightly, 3 To some extent, 4 Quite a lot 5 Very much

### Respondents

The questionnaire was sent six times to 77 employees and managers who worked at the studied office. The number of respondents was 47, 37, 33, 30, 28, and 28 in *conditions* P1a, N1, N2, N3, N4, and P1b, respectively. The response rates were 61, 48, 43, 39, 36, and 36%. Most of the respondents worked at the office daily. The mean age of eighteen employees (18) who responded in all *conditions* was 38 years. The standard deviation was 11 years. The percentage of female respondents was 56. The mean age of non-respondents was 45 years (13% female). Thus, the core group of 18 employees was slightly over-represented by young female persons. The analyses were conducted on this group only (within-subjects design) so each of them acted as his/her own control. This gives more reliable results than the other alternative where all responded employees are taken into account (between-groups analysis). Similar approach was also applied in a recent field experiment (Hongisto et al., 2016a).

### Statistical methods

The analyses were conducted using IBM SPSS Statistics version 23 (Armonk, NY: IBM Corp). The differences in the measured *SPL*s between *conditions* were tested using *t*-test for independent samples (two-tailed). The analyses of the subjective responses between *conditions* P1a–N4 (five *conditions*) were made using repeated measures ANOVA with the *condition* as a within-subject variable. When a main effect was found, paired comparisons between *conditions* were performed using *t*-tests. An alpha level of 0.05 was used in all analyses. Whenever needed, the homogeneity of variance was estimated with Mauchly's test of sphericity. When the test indicated a violation of sphericity, the Greenhouse-Geisser correction was applied and the corresponding *p*-values are reported. It should be noted that the number of employees was not 18 for all tests because every employee did not necessarily respond in all questions in all six *conditions*. The Benjamini-Hochberg procedure (Benjamini and Hochberg, 1995) was used for alpha-error adjustment in paired comparisons. The difference between *conditions* P1a and P1b was tested using paired samples *t*-test (two-tailed). Effect size describes the strength of the difference between two conditions 1 and 2. The effect size is large if *d* > 0.8, medium if *d* > 0.50 and small if *d* > 0.20. Effect size was determined by Cohen's *d* according to Lakens (2013)

$$\begin{array}{l} d=\frac{\left|M_{1}\;-\;M_{2}\right|}{\frac{1}{2}\left(s_{1}\;+\;s_{2}\right)} \end{array}$$

where *M*_i_ and s_i_ are the mean and the standard deviation of condition i, respectively.

## Results

### Quantitative results

The measured sound pressure levels of the masking sounds in each *condition* are shown in Table 1 and Figure 1. The A-weighted levels of the *conditions* differed significantly from each other (*p* < 0.001). The *SPL* of every third octave frequency band differed significantly from each other (all *p*'s under 0.001). However, the difference is negligible from subjective point of view (see Discussion).

Perceived disturbance caused by various environmental factors varied strongly between *conditions* P1a–N4 (Table 4). The largest changes were observed in *noise* (see the definitions of variables in Table 2). There was a significant main effect of *condition* (P1a–N4) on *noise* (*F*_4,56_ = 5.65, *p* < 0.001, η^2^ = 0.29). Paired comparisons showed significant differences between *conditions* P1a and N3 (*p* < 0.001, *d* = 1.50), *conditions* P1a and N4 (*p* < 0.001, *d* = 1.34), *conditions* N1 and N3 (*p* < 0.05, *d* = 0.61), and *conditions* N1 and N4 (*p* < 0.05, *d* = 0.50). Disturbance by *noise* was also higher in the *condition* P1b than in P1a [*t*_(17)_ = −3.37, *p* < 0.01, *d* = 0.72]. A significant main effect of *condition* (P1a–N4) was also found for *lack of speech privacy* [*F*_(4, 68)_ = 3.19, *p* < 0.05, η^2^ = 0.19] and *movements in vision* [*F*_(4, 64)_ = 2.58, *p* < 0.05, η^2^ = 0.14]. The paired comparisons did not reveal differences between individual *conditions*. Comparison between *conditions* P1a and P1b revealed also significant differences between *cold* [*t*_(17)_ = 2.38, *p* < 0.05, *d* = 0.59] and *crowdedness* [*t*_(17)_ = −2.47, *p* < 0.05, *d* = 1.17].

**Table 4:** Perceived disturbance by various environmental factors.

**Environmental factor**	***Condition***							
	**P1a**	**N1**	**N2**	**N3**	**N4**	**P1b**	***p*_1_**	***p*_2_**
*Draught*	1.9	1.4	1.7	2.1	1.8	1.7	n.s.	n.s.
*Cold*	2.4	1.7	1.8	2.3	2.1	1.8	n.s.	<0.05
*Hot*	2.4	2.3	2.3	2.4	2.0	1.9	n.s.	n.s.
*Stuffy air*	1.5	1.5	1.6	1.9	1.5	1.6	n.s.	n.s.
*Noise*	2.1	2.9	3.0	3.7	3.5	2.8	<0.001	<0.01
*Lack of speech privacy*	2.1	2.7	3.0	3.3	3.0	2.6	<0.05	n.s.
*Lighting*	1.4	1.4	1.2	1.5	1.5	1.6	n.s.	n.s.
*Glare or reflections*	1.5	1.1	1.2	1.4	1.4	1.8	n.s.	n.s.
*Dust or dirt*	1.4	1.1	1.2	1.2	1.1	1.2	n.s.	n.s.
*Smells*	1.2	1.0	1.0	1.2	1.2	1.3	n.s.	n.s.
*Disorder*	1.3	1.3	1.2	1.3	1.2	1.5	n.s.	n.s.
*Crowdedness*	1.0	1.1	1.1	1.4	1.3	1.5	n.s.	<0.05
*Movements in vision*	1.7	1.2	1.4	1.7	1.4	1.7	<0.05	n.s.
*Openness*	1.3	1.2	1.4	1.7	1.6	1.5	n.s.	n.s.

*Mean values. Scale: 1 Not at all, 5 Very much. Column p_1_ describes the level of significance of the main effect between conditions P1a-N4. Column p_2_ describes the level of significance between conditions P1a and P1b*.

**Figure 5:**
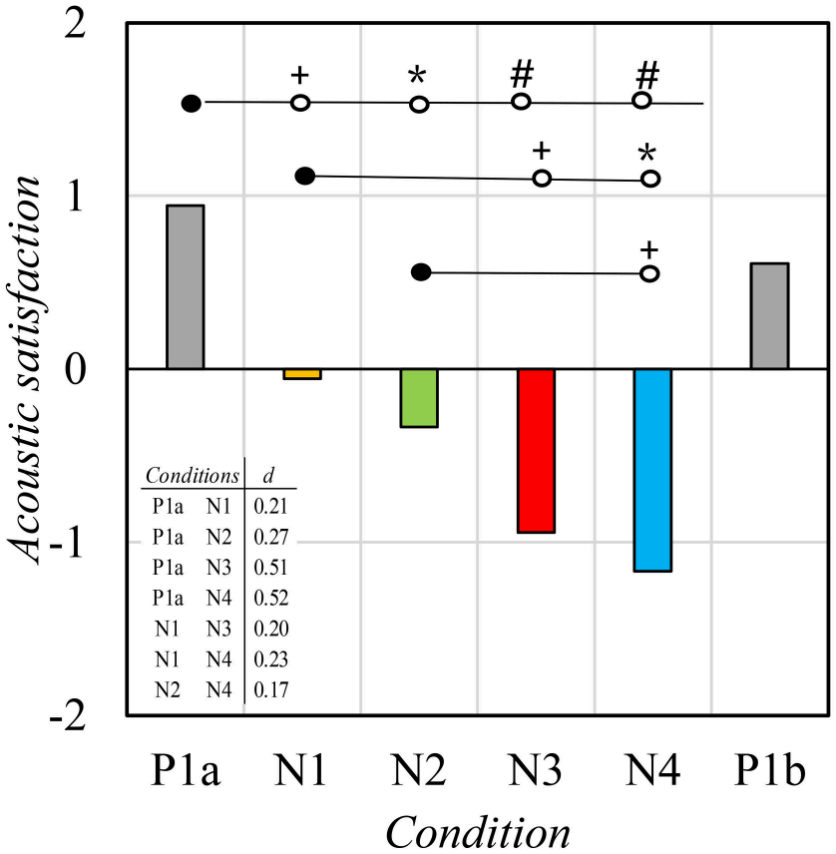
The mean satisfaction with the noise and acoustic environment of the office as a whole. Scale: −2 very dissatisfied; +2 very satisfied. Levels of statistical significance: +*p* < 0.05, ^*^p < 0.01, #*p* < 0.001.

The *condition* (P1a–N4) affected the *acoustic satisfaction* [*F*_(4, 68)_ = 12.94, *p* < 0.001, η^2^ = 0.43]. Paired comparison indicated that *acoustic satisfaction* reduced systematically when a new *condition* was presented (Figure 5). The *condition* P1a was rated as the best *condition* followed by N1. *Conditions* P1a and P1b did not differ from each other suggesting that *acoustic satisfaction* normalized when the original masking sound was re-introduced.

**Figure 6:**
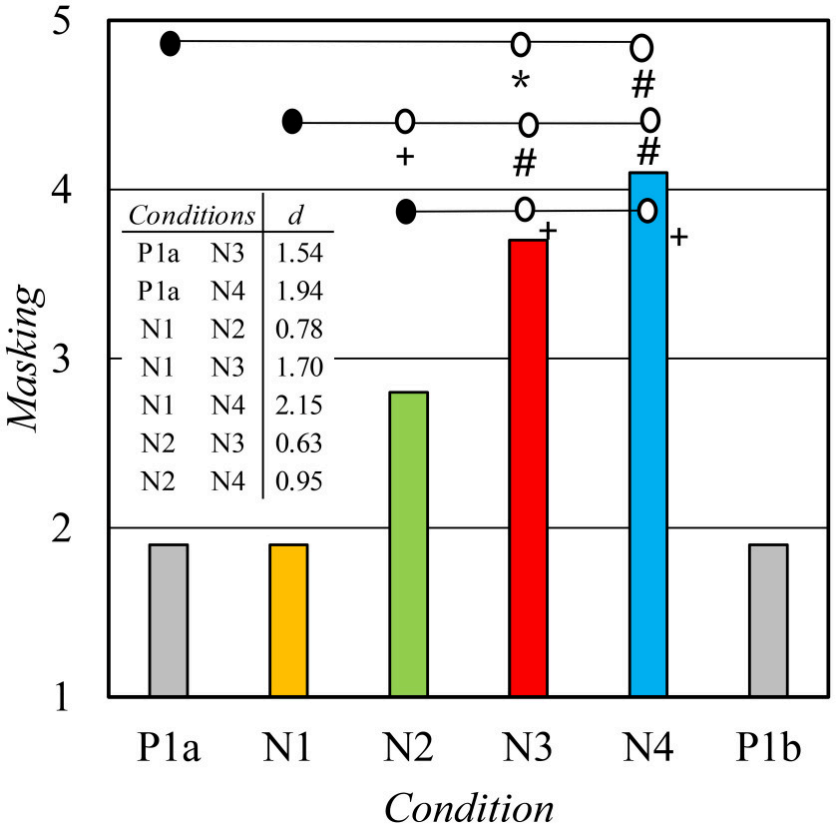
The mean disturbance of work caused by sound masking. Scale: 1 Not at all, 5 Very much. Levels of statistical significance: +*p* < 0.05, ^*^p < 0.01, #*p* < 0.001.

Perceived disturbance caused by various sound sources varied strongly between *conditions* (Table 5). A significant main effect of *condition* (P1a–N4) was found for most of the sound sources. Significant differences were not observed for any sound source between *conditions* P1a and P1b. Paired comparisons are reported below for three relevant sound sources. Differences were statistically significant for *nearby speech* [main effect: *F*_(4, 68)_ = 3.94, *p* < 0.05, η^2^ = 0.19] between *conditions* P1a and N3 (*p* < 0.05, *d* = 1.16) and between *conditions* N1 and N3 (*p* < 0.05, *d* = 0.54). Differences were statistically significant for *remote speech* [main effect: *F*_(4, 68)_ = 4.80, *p* < 0.01, η^2^ = 0.22] between *conditions* P1a and N3 (*p* < 0.05, *d* = 1.16), *conditions* P1a and N4 (*p* < 0.05, *d* = 1.05) and *conditions* N1 and N3 (*p* < 0.05, *d* = 0.58). Differences were statistically significant also for *masking* [main effect: *F*_(4, 68)_ = 14.48, *p* < 0.001, η^2^ = 0.46]. All possible paired comparisons between *conditions* P1a–N4 for *masking* are shown in Figure 6.

**Table 5:** The perceived disturbance of work by different sound sources.

**Sound source**	***Condition***							
	**P1a**	**N1**	**N2**	**N3**	**N4**	**P1b**	***p*_1_**	***p*_2_**
*Nearby speech*	2.1	2.7	2.5	3.3	2.9	2.2	<0.05	n.s.
*Remote speech*	1.5	2.2	2.1	2.9	2.8	1.7	<0.01	n.s.
*Ventilation*	1.7	1.4	1.8	2.4	2	1.6	<0.05	n.s.
*Masking*	1.9	1.9	2.8	3.7	4.1	1.9	<0.001	n.s.
*Work sounds*	1.4	2.1	2.2	2.4	2.5	1.6	<0.05	n.s.
*Equipment*	1.2	1.7	1.8	2.1	1.9	1.2	<0.05	n.s.
*Phones*	1.2	1.4	1.3	1.4	1.5	1.2	n.s.	n.s.

*Mean values. Scale: 1 Not at all, 5 Very much. Columns p_1_ and p_2_ follow the Table 4*.

The perceived effects of office sounds on performance varied significantly between *conditions* P1a–N4 (Table 6). Paired comparisons showed that *conditions* N3 and N4 differed significantly from *condition* P1a for all types of work (*p*'s below 0.01 or below 0.05, *d*'s between 1.01 and 1.39). Significant difference was also found between *conditions* P1a and N1 (*cooperation, p* < 0.05, *d* = 0.59), and *conditions* P1a and N2 (*telephone conversations, p* < 0.05, *d* = 0.41).

**Table 6:** The perceived effect of sounds on performance in different tasks.

**Type of work**	***Condition***							
	**P1a**	**N1**	**N2**	**N3**	**N4**	**P1b**	***p*_1_**	***p*_2_**
*Reading and writing*	1.6	2.0	1.8	2.7	2.7	1.6	<0.001	n.s.
*Planning*	1.4	2.2	1.9	2.9	2.8	1.8	<0.001	n.s.
*Phone conversations*	1.7	2.2	2.3	3.2	2.8	1.9	<0.001	n.s.
*Telemeetings*	1.6	2.0	2.0	3.1	2.9	1.7	<0.001	n.s.
*Cooperation*	1.3	1.8	1.7	2.7	2.6	1.6	<0.001	n.s.
*Confidential discussions*	1.3	1.6	1.7	2.7	2.3	1.4	<0.001	n.s.

*Mean values. Scale: 1 Not at all, 5 Very much. Columns p_1_ and p_2_ follow the Table 4*.

The sound quality of masking was inquired by the same nine attributes which were used in the listening test prior to this experiment. A significant main effect of the *condition* (P1a–N4) on all sound quality attributes was found (Table 7). Paired comparisons showed a large number of differences between the *conditions*. We were content to report the differences between *condition* P1a and other *conditions*.

**Table 7:** Mean values of the attributes describing the quality of sound masking.

**Attribute**	***Condition***							
	**P1a**	**N1**	**N2**	**N3**	**N4**	**P1b**	***p*_1_**	***p*_2_**
*Easy to habituate to*	1.4	0.8	−**0.2**	−**1.8**	−**2.2**	1.4	<0.001	n.s.
Distracting^*^	−1.2	−1.1	−0.1	**1.3**	**1.3**	−1.5	<0.001	n.s.
*Pleasant*	−0.6	−0.7	−1.2	−**1.9**	−**2.3**	0.2	<0.001	n.s.
Stressful^*^	−1.8	−1.2	−0.9	**0.1**	**0.8**	−1.7	<0.001	n.s.
*Natural*	−0.9	−0.7	−1.1	−1.8	−**2.3**	−0.5	<0.05	n.s.
Annoying^*^	−1.9	−**0.9**	**0.1**	**1.0**	**2.3**	−1.3	<0.001	n.s.
Tiring^*^	−1.9	−**1.0**	−**0.7**	**0.2**	**1.1**	−1.4	<0.001	n.s.
*Acceptable*	0.7	0.0	−**0.7**	−**1.8**	−**2.3**	1.2	<0.001	n.s.
*Helpful for my work*	0.3	−0.2	−**1.2**	−**1.6**	−**2.4**	0.6	<0.001	n.s.

Scale: −3 Strongly disagree, +3 Strongly agree. Bold font means that the condition differs significantly from condition P1a. Large value is desirable except for negative attributes labeled with asterisk ^*^.

The perception of the *adequacy of masking level* varied between *conditions* P1a–N4 [*F*_(4, 68)_ = 3.23, *p* < 0.05, η^2^ = 0.16]. The level of masking sound was rated the most adequate in *conditions* P1a and N1. However, paired comparisons did not show significant differences between *conditions*.

**Figure 7:**
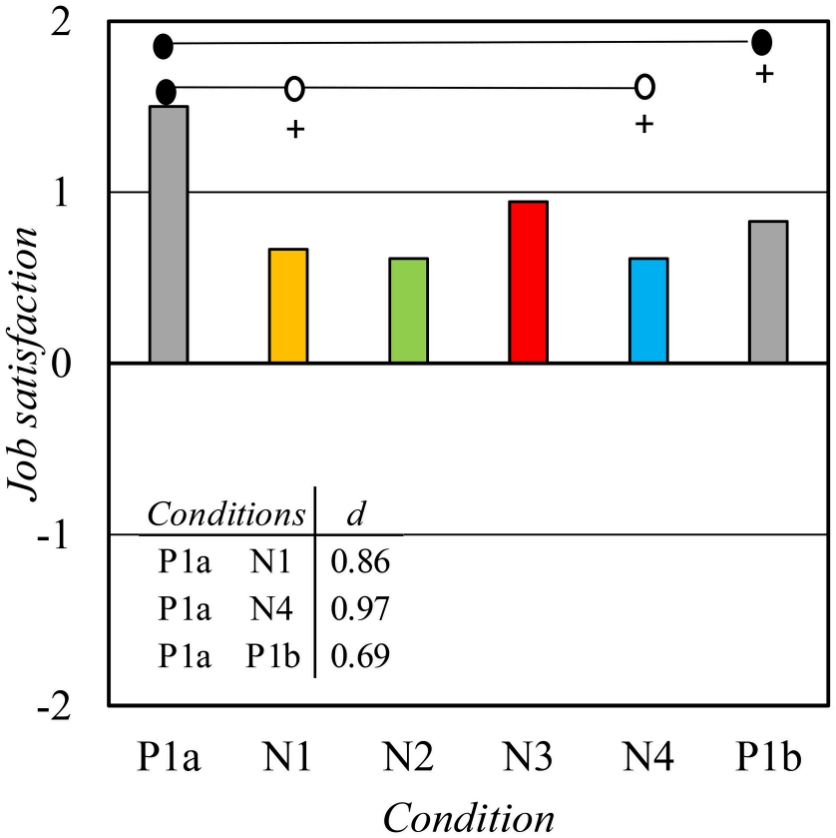
The mean satisfaction with work as a whole. Scale as in Figure 4. Symbol + means that the *condition* differs significantly from P1a (*p* < 0.05).

A main effect of *condition* was also observed on *job satisfaction* [*F*_(4, 68)_ = 3.98, *p* < 0.05, η^2^ = 0.19]. A significant and almost permanent decrement in *job satisfaction* was observed after the *condition* P1a (Figure 7). Interestingly, *job satisfaction* did not return to the baseline level (P1a) after the experiment even though the other perceptions generally did. *Condition* did not have a significant main effect on *stress*: the mean values varied between 2.33 and 2.83 without any clear trend. *Possibilities to influence* [*t*_(16)_ = 2.73, *p* < 0.05, *d* = 0.14] was significantly lower in *condition* P1b (mean 2.8) than in *condition* P1a (mean 3.3). *Social support* did not change significantly from P1a to P1b.

### Qualitative results

Responses to the open question (see Table 2) are summarized below. The information can be used to complement the quantitative results. Overall, sound masking was desired to be adaptive to the changes in the sound level of the office. In addition, some respondents desired the sound masking to be adapted to varied work requirements in different teams.

During *condition* P1a, both positive and negative opinions were given. *Condition* P1a was described to sound like an aircraft or air-conditioning. During *condition* N1, many comments reported that others' conversations could be heard. Some respondents told they were working at home or other spaces because of the noisy environment. During *condition* N2, sound masking was described as distracting and unpleasant/awful and conversations could be heard. Sound was described as, e.g., public toilet, fan and running water. *Condition* N3 was described as distracting, unpleasant/awful and some felt more stressed. Sound masking was not perceived to mask sounds and conversations could be heard. Sound was described as “problem with a ventilation system” and “toilet is constantly running.” *Condition* N4 was described as annoying and distracting and some respondents told they were working at home or other spaces because of the noisy environment. In addition, sound masking was not perceived to mask sounds and conversations could be heard. Sound was described as “a fish tank,” “bath running upstairs,” and “raining on a tin roof.” *Condition* P1b was found less distracting than conditions N1–N4 and better than sounds during past few months. However, some respondents found that sound masking level was too low. Only one respondent mentioned that this sound was equal with the first sound.

## Discussion

### Sound pressure levels

The A-weighted *SPL, L*_Aeq_, of masking sound varied significantly between the *conditions* although large effort was made to achieve identical levels. The mean values varied between 43.0 and 44.6 dB. Fortunately, humans are usually not able to detect A-weighted level differences less than 1.0 dB. For example, Oliva et al. (unpublished) found that the difference in the loudness, or the annoyance, of two spectrally identical wide-band sounds can reach statistical significance if the level difference is 2 dB or larger. It should also be noted that *conditions* P1a and P1b differed by 0.8 dB with respect to *L*_Aeq_ although the electronic settings of the sound masking system were equal. Differences appeared only at certain frequency bands (see Figure 1) and we cannot explain them. This finding suggests that the measurement uncertainty of *L*_Aeq_ for each *condition* can be up to 1 dB. Therefore, we can relatively safely conclude that the A-weighted levels of the *conditions* were nearly equal and that the differences in *L*_Aeq_ hardly explain the differences in subjective ratings between the *conditions*.

### Environmental perception

The results suggest that the type of masking sound can have an influence on subjective outcomes; conventional pseudo-random masking sound, i.e., the *conditions* P1a and P1b, produced the best perceived working conditions. This conclusion is supported by the following chain of findings:

- Perceived disturbance by *noise* and the disturbance of work caused by main sound sources (*nearby speech, remote speech, masking*) suggests that *conditions* N1 and N2 were nearly similar with P1a but *conditions* N3–N4 were not.
- *Acoustic satisfaction* suggested that *conditions* N1–N4 were worse than P1a.
- Perceived effects of sounds on performance suggested that *conditions* N3 and N4 were worse than P1a.
- *Conditions* N2–N4 were worse than *condition* P1a because more than half of the attributes of sound masking differed significantly from *condition* P1a to the adverse direction.
- Taking all subjective variables into account, none of WBMSs were ever better than the pseudo-random masking sound (P1a or P1b).

The overall picture supports the view that the employees were much more satisfied with their original masking sound P1a than the WBMSs N1–N4. It is notable that the *condition* N1 was similar to P1a in terms of several subjective measures. This may be explained by the fact that the N1 spectrum did not differ very much from P1a. Therefore, future research attempting to find an adequate WBMS is justified.

The WBMSs N1–N4 differed significantly from each other with respect to spectrum (Figure 1) and the type of water flow (Table 1). Therefore, it cannot be ruled out that the masking sounds itself produced the significant differences in the subjective ratings between *conditions*. This is supported by the finding of Galbrun and Ali (2013): the sound quality of water flow, which depends on various physical factors (such as flow rate, height, and width of water flow), had a strong influence on objective sound quality (overall level, spectrum, roughness, sharpness, pitch strength), and on the subjective perception (peaceful, relaxing) of water sounds, when played together with road traffic noise with varying signal-to-noise ratios.

The WBMSs N1–N4 were selected from a listening test using stereo recordings and stereo headphones. The sounds were pleasant and natural and based on stereo recordings. Stereo recordings are usually made by using two microphones (Left and Right) separated by 20 cm. As a consequence of this, the two signals (Left and Right) differ slightly with respect to phase and spectrum. However, the sound masking system installed in the office could only play monophonic signals. When the first water sound was launched, we observed that the sound was much less spacious than during headphone listening because all loudspeakers played exactly the same signal without a phase difference, contrary to stereo headphone listening. A similar difference in spatial sensation can be experienced by changing stereo playback to mono playback using stereo loudspeakers or headphones. In addition, the frequency response of the loudspeakers, i.e., the output level in different frequencies, was less flat than the response of headphones used in the listening test. These factors biased the water-based masking signals making them to sound a clearly different compared to sensation during headphone listening.

Some new commercial sound masking systems allow the playback of two-channel signals (stereo) so that the two channels (Left and Right) are fed by independent signals with a small phase difference between them. The two channels are distributed evenly in the masking loudspeakers of the office and the sound environment gets more spacious because of the phase differences from nearby loudspeakers. Future research is welcome to find out whether the playback mode (Mono or Stereo) affects the perception of sound masking.

The employees reported that *hot* and *cold* had the largest disturbing effect on working during condition P1a. *Noise* and *lack of speech privacy* are usually more disturbing factors of work environment in open-plan offices (Pejtersen et al., 2006; Haapakangas et al., 2008; Bodin Danielsson and Bodin, 2009; Frontczak et al., 2012). It seems that that the perceived acoustic conditions were not especially bad in the workplace prior to the experiment. This finding gives indirect support for the use of pseudo-random masking sound, i.e., *conditions* P1a and P1b. Instead, perceived *noise* was significantly higher in *condition* P1b than in P1a. Unlike many other sound-related variables, perceived *noise* did not return to the baseline level after the experiment. There may be several explanations for this. The increased *noise* in P1b may suggest that the respondents reacted negatively to the experiment which had lasted over 3 months when the responses for P1b were gathered. It is also possible that the activity in the office, and the related activity noise levels, was higher during condition P1b (winter) than during condition P1a (early fall). We did not measure the activity noise levels because the office was large: reliable monitoring of office noise would have required at least 10 measurement points. Such a large investment was beyond the resources of the research project. The findings demonstrate how difficult it is to control complicated environmental factors, such as office noise, in long-term field experiments.

The results can also be interpreted from another perspective. Because all *conditions* had nearly equal A-weighted *SPL*, 43.0–44.6 *L*_Aeq_, the results suggest that the A-weighted *SPL* of masking is not the only objective descriptor that defines the masking efficiency and subjective perception. This is in line with the laboratory experiment of Haapakangas et al. (2011) which showed that the type of masking sound had an effect on cognitive performance and acoustic satisfaction even though the A-weighted *SPL* was constant. Additionally, Hongisto et al. (2015) found that the spectrum alone explained the acoustic satisfaction of pseudo-random noises. Our study supports the findings of these previous studies but we cannot say which is more important: the spectrum or the type of sound.

It is possible that the perception of water sounds could be explained to some extent by more sophisticated sound quality descriptors, which take, e.g., the temporal variation or spectral envelope into account. For example, Jeon et al. (2010) found that sharpness, which describes the spectral envelope of sound, affected the perception (preference and semantic differential test) of water sounds while played together with road traffic noise. Although their study was beyond the context of our study for many reasons (design, study environment, primary sound, dependent variables, sound levels), it may be useful to take these parameters into account while designing future experiments. In our study, *conditions* N2 and N3 had a stronger high frequency content than the other conditions, thus representing larger sharpness. These features are expected to reduce preference according to Jeon et al. (2010) and acoustic satisfaction according to Hongisto et al. (2015). However, the subjective ratings of our study did not support abovementioned expectations based on laboratory experiments. Our research question was not designed to enable a systematic analysis between subjective responses and sound quality descriptors, such as sharpness, loudness or fluctuation strength (Jeon et al., 2010). In addition, field studies are sensitive to several non-acoustic factors which are not present in laboratory experiments. Therefore, our field study cannot provide any conclusions regarding the role of sound quality descriptors.

It should be noted that water-based sound contain more information than pseudo-random noise because of the temporal variation and the inevitable association to water flow which is not physically present. The changing state hypothesis (Jones et al., 1992) suggests that the automatic processing of temporally varying sounds activates the same processes that are required in maintaining order information in short-term memory. WBMSs can attract bottom-up attention more easily and as such distract from the task at hand. This can result in lower appreciation of the water sound.

Unpublished information obtained from a case study in another open-plan office suggests that employees were satisfied with WBMS in an open-plan office where visual cues of water were strongly present (Benway, unpublished). Masking was presented at a level of 45–48 dB *L*_Aeq_ via loudspeakers in remote parts of the office where the direct sound from the waterfall could not reach. The office involved a waterfall and visual cues of the waterfall were present also in other areas of the office. A laboratory study by Haga et al. (2016) has shown that the subjective effects of an ambiguous water-like sound do not depend on the stimulus-features *per se* but on the interpretation of the sound. Water sound may have been too artificial in our experiment because the environment around the building did not involve water elements. Thus, the water sound may have rather evoked negative interpretations, such as a running toilet, as shown by the open responses.

It may be useful to mention the laboratory experiment of Jeon et al. (2010) where road traffic noise at 55 dB *L*_Aeq_ was masked with very different water sounds at 55 dB while different pictures were shown to the participants. They found that the percentage of water features in the pictures was significantly correlated with improvements in the preference score between audio-only and audio-visual sessions. Because Haapakangas et al. (2011) and Keus van de Poll et al. (2015) supported WBMSs and the *condition* N1 involving a WBMS did not differ from condition P1a (pseudo-random masking sound) for every variable, it would be useful to investigate how the visual cues affect the perception of water sounds compared to the absence of visual cues.

### Job satisfaction

*Job satisfaction* reduced after the first *condition* and it did not return to the baseline level at the end in the same way as several subjective variables related to the acoustic environment did. *Job satisfaction* measures the overall satisfaction with work being a significantly broader variable than any other subjective variable of this study. Our experiment involved a strong and an obvious manipulation of the work environment. It is possible that *job satisfaction* was reduced because of negative changes in *acoustic satisfaction* and several other related variables. Prior to the experimental conditions (P1a), *lack of privacy* and *noise* were the most adverse factors of the work environment. Negative changes in them were observed right after the first water-based masking *condition* N1. This change might be sufficient to trigger a reduction in *job satisfaction*.

The experiment itself, and its management, could be another reason for the reduction in *job satisfaction*. Hongisto et al. (2016a) showed that job satisfaction improved in an open-plan office after a holistic refurbishment of indoor environment. Significant positive changes were found in almost all factors related to the perception of the physical work environment. The authors suspected that a user-oriented, appropriate design of the refurbishment, strong demand for environmental improvements by the employees and professional change management contributed to the positive findings. Our experiment did not include the effects of user-involvement. The lack of control over acoustic changes may have contributed to the dissatisfaction with the new water-based sounds. It is possible that the employees did not find the experiment useful because they already had a sound masking system.

Earlier research has shown that increased perception of control over the work environment is associated with higher environmental satisfaction (O'Neill, 1994; Veitch and Gifford, 1996; Huang et al., 2004; Lee and Brand, 2010). In laboratory experiments, which usually do not last more than a couple of hours, subjects may accept the experimental conditions more easily than at workplaces. In addition, employees may be engaged with the workplace and many of them wish to influence matters that concern their daily environment. In our study, employees did not have a chance to affect the *conditions*, which probably reduced the perceived control of work environment. This is also supported by the finding that *possibilities to influence* was perceived lower after the experiment. It seems that the employees did not find personal or other benefit from this study and they felt that the study only interfered their work.

It is also possible that other negative changes occurred in the workplace during the experimental period which explained the results.

### Method

The methodological strengths of our field study were that the experimental conditions, i.e., the sound masking spectra and level, were reported in detail and carefully realized and measured. Second, we returned to the *condition* P1a in the end of the experiment so that we could better compare the WBMSs to pseudo-random masking sound. Third, the employees were accustomed to work under artificial masking sound prior to the experiment so that the perceptions of sound masking were probably more reliable than in an opposite situation.

The methodological weaknesses of our study include the lack of a control group, the predefined order of conditions, the large number of experimental conditions (length of the experiment), the drop-out rate of respondents with time, lack of “masking off” condition, and bias toward the original masking sound of *condition* P1a.

The lack of a control group could be seen as a methodological weakness which prevents the inference of causality. However, using a control group was not possible in this case because all office employees were subject to the experiment. Choosing a control group from another unit or company would not have been meaningful because the group would not have been comparable in terms of the work environment, especially sound masking, or working style. A long-term survey may also create expectations of problem abatement in the control group, and may exacerbate the perceived problems when the survey is repeated six times without any sign of benefiting from it. Thus, a control group might not work as an intended neutral point of comparison in this kind of complicated experimental research. For the same reason, it is difficult to establish good co-operation with the manager of a control group which would be vital for sufficient response rates. It should be noted that conditions P1a and P1b acted as neutral points of comparison and the differences between them were mainly insignificant, what comes to the perception of sound masking.

The order of conditions is usually completely randomized between subjects in laboratory experiments following repeated measures design. Unfortunately, random assignment to conditions may be impossible to realize in field settings where all employees are exposed to the same conditions. Future workplace experiments should probably contain fewer subsequent sound conditions to avoid order effects and the related reduction of motivation and response rate. The control condition, here P1, could follow each experimental condition N, e.g., P1-N1-P1 and P1-N2-P1 and not P1-N1-N2-P1. Alternatively, a cross-over design might be applied where the employees are divided into two groups exposed to different orders: P1-N1-N2-P1 and P1-N2-N1-P1. A smart cross-over design was applied by, e.g., Seddigh et al. (2015). In addition, the selected masking sounds should be based on careful listening tests in laboratory conditions before submitting to a workplace. In our case, the listening experiment was conducted via the internet without controlling the type of headphones or listening levels so that we did not report the results in detail.

A masking off condition was excluded because the employees had been used to the sound masking of *condition* P1a. The managers did not find it adequate to turn the sound masking off because they believed it would reduce work performance and environmental satisfaction. The problem with masking off condition is also that it is not well-defined in scientific terms. In the office of our study, masking off condition would mean a level of 40 dB *L*_Aeq_ (ventilation of Figure 1) which is much higher than the masking off conditions in prior field studies (Helenius and Hongisto, 2004; Hongisto, 2008; Hongisto et al., 2012), or the silent conditions of laboratory experiments (Venetjoki et al., 2006; Haka et al., 2009; Haapakangas et al., 2011, 2014). Therefore, the inclusion of masking off condition with a level of 40 dB might have led to misleading results.

It should be noted that the employees were very familiar with the original sound masking prior to the experiment (*conditions* P1a and P1b). The high familiarity could explain the strong preference of them. It may take much more than 3 weeks to get accustomed with a new masking sound. Unfortunately, we cannot recommend a time after which the familiarization has reached a sufficient level.

Finally, our experiment was conducted at an individual workplace. Different results might emerge from another workplace. Therefore, our results cannot be generalized but can, merely, be used as a reference for designing future investigations.

Further research in this field is important because a recent field study showed that noise disturbance is higher in offices where the distraction distance is large (Haapakangas et al., 2017). It was also found that the background noise level of the office (noise level of masking and ventilation) was associated with noise disturbance: the lower the background noise level was, the higher was the perceived noise disturbance. The best way of producing adequate background noise level is artificial sound masking because dense grid of loudspeakers allows the minimization of spatial differences in sound level. Modern systems can also adapt according to the ambient office noise. Therefore, further research on adequate and user-oriented sound masking technologies (sound types, playback systems, sound levels, spectra and control over the level) are strongly supported.

### Comparison to other field studies

Our study is unique because careful field studies in this research area have not been published very much. A couple of field studies have investigated the change from the office without sound masking to an office with pseudo-random masking sound, such as P1a, using before-after design (Helenius and Hongisto, 2004; Hongisto, 2008; Hongisto et al., 2012). These studies gave some support for the use of pseudo-random masking sound when the *SPL* was approximately 43 dB *L*_Aeq_ and the spectrum was close to −5 dB per octave doubling (see Figure 1).

## Conclusions

We tested how four different WBMSs were perceived in an open-plan office compared with conventional pseudo-random masking sound. The employees were used to the pseudo-random masking 2 years prior to the experiment so the experiment was not influenced by the unfamiliarity of sound masking concept. All masking sounds were presented nearly at the same equivalent sound pressure level, 43–45 dB *L*_Aeq_ but the spectrum and the type of water flow differed. The results were not in favor of WBMSs: none of the WBMS produced better outcomes than pseudo-random noise in any investigated subjective variable. The study had some methodological and audio-technical shortcomings so we cannot conclude that water-based sounds would not be adequate masking sounds at workplaces. Some subjective variables showed that the first WBMS, which had exactly the same spectrum as the pseudo-random masking sound, was perceived to be equal with the pseudo-random masking sound. The results give space for further research in this topic especially because the use of sound masking is one of the most efficient ways to reduce the distraction caused by colleagues' speech.

## Author contributions

VH has written the manuscript entirely. JV has conducted the questionnaires and statistical analyses. DO has conducted the measurements and sound preparations. EB has managed the project together with VH. All authors have given valuable comments on the manuscript. VH has written the revised manuscript. JV and AH have adviced in the revised manuscript regarding statistical analyses.

### Conflict of interest statement

EB is affiliated with a commercial company, Plantronics Inc., USA. The other authors declare that the research was conducted in the absence of any commercial or financial relationships that could be construed as a potential conflict of interest.
